# Speed Switching of Gonococcal Surface Motility Correlates with Proton Motive Force

**DOI:** 10.1371/journal.pone.0067718

**Published:** 2013-06-24

**Authors:** Rainer Kurre, Nadzeya Kouzel, Kanimozhi Ramakrishnan, Enno R. Oldewurtel, Berenike Maier

**Affiliations:** Department of Physics and Biocenter, University of Cologne, Köln, Germany; University of Groningen, Netherlands

## Abstract

Bacterial type IV pili are essential for adhesion to surfaces, motility, microcolony formation, and horizontal gene transfer in many bacterial species. These polymers are strong molecular motors that can retract at two different speeds. In the human pathogen *Neisseria gonorrhoeae* speed switching of single pili from 2 µm/s to 1 µm/s can be triggered by oxygen depletion. Here, we address the question how proton motive force (PMF) influences motor speed. Using *pHluorin* expression in combination with dyes that are sensitive to transmembrane ΔpH gradient or transmembrane potential ΔΨ, we measured both components of the PMF at varying external pH. Depletion of PMF using uncouplers reversibly triggered switching into the low speed mode. Reduction of the PMF by ≈ 35 mV was enough to trigger speed switching. Reducing ATP levels by inhibition of the ATP synthase did not induce speed switching. Furthermore, we showed that the strictly aerobic *Myxococcus xanthus* failed to move upon depletion of PMF or oxygen, indicating that although the mechanical properties of the motor are conserved, its regulatory inputs have evolved differently. We conclude that depletion of PMF triggers speed switching of gonococcal pili. Although ATP is required for gonococcal pilus retraction, our data indicate that PMF is an independent additional energy source driving the high speed mode.

## Introduction

Bacterial type IV pili (T4P, pili) are extracellular polymers that are generated by various bacterial species [1]. They are involved in adhesion to surfaces, motility, microcolony formation and biofilm architecture, and in transformation.

**Figure 1:**
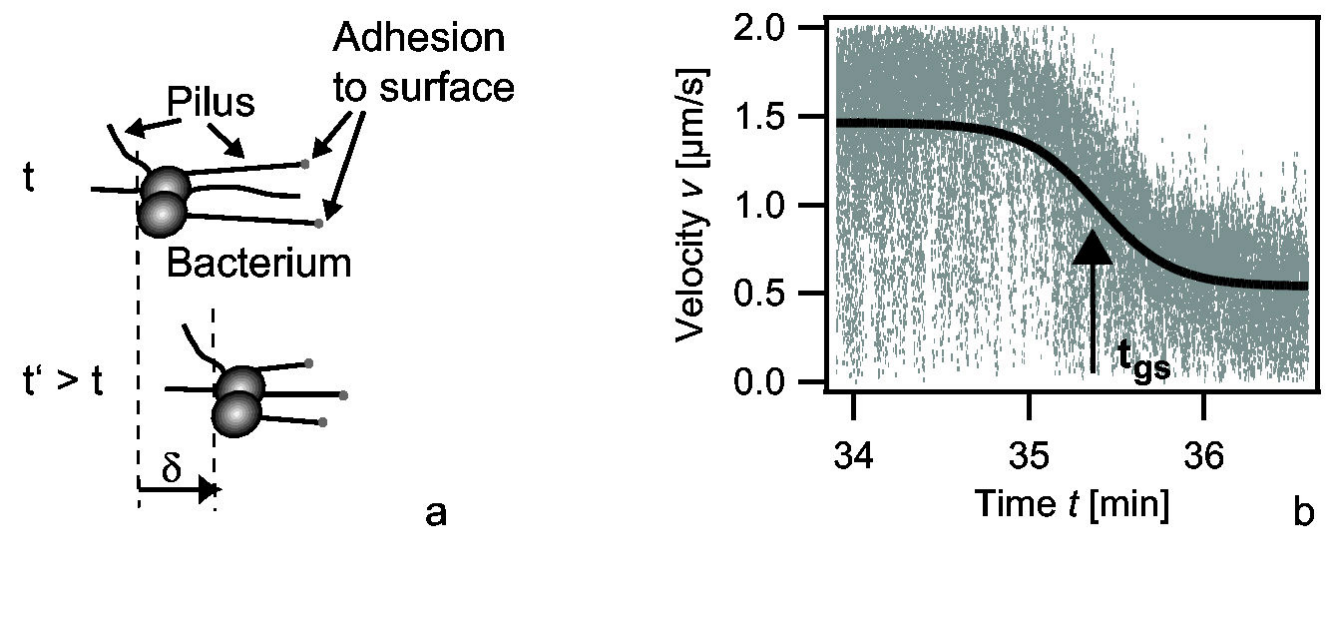
Oxygen depletion triggers speed switching of T4P retraction. a) Scheme of T4P driven surface motility. Multiple pili adhere to the surface and when they retract, they pull the cell towards the point of attachment. b) Overlay of the speed of twitching motility of multiple bacteria during global speed switching. Full line: fit to eq. 1.

The type IV pilus mainly consists of pilin subunits that assemble to form helical polymer with a width of 6 nm and an average length of 1 µm [2]. The length of T4P is dynamic, i.e. pili elongate by polymerization and retract by depolymerization [3,4]. The ATPase PilF is essential for polymerization of pili [5] and the ATPase PilT is essential for pilus retraction in *Neisseria gonorrhoeae* (*N. gonorrhoeae*, gonococcus) [6]. Both ATPases form hexameric rings and structural data suggests coordinated ATPase cycles of the individual motors in the ring [7]. Cycles of pilus elongation, adhesion at surfaces, and retraction power bacterial surface motility, also called twitching motility. Multiple T4P cooperate for generating surface motility (Figure 1a) [8]. During retraction, single pili can generate considerable force exceeding 100 pN [9]. Potential functions of high force generation include the rearrangement of the host cytoskeleton [10–12] and force-induced change of epitope exposure on the T4P [13].

The physical parameters of T4P retraction can be fine-tuned [14]. At the genetic level, PilT2 enhances the speed of T4P retraction [15]. We have recently shown that type IV pili of *N. gonorrhoeae* can switch between different velocities, namely retraction at two different speed modes and elongation [16–18]. Speed switching is conserved in *Myxococcus xanthus* [19]. For *N. gonorrhoeae* we found that oxygen depletion triggers the switch from the high speed mode of single pilus retraction at v_H_ ≈ 2 µm/s to the low speed mode at v_L_ ≈ 1 µm/s [20]. Switching occurred at the level of individual pili, was reversible, and independent of protein expression. Twitching motility of gonococci exhibits a global switch from a high speed mode of surface motility v = 1.5 µm/s to a low speed mode v = 0.5 µm/s upon oxygen depletion [20] (Figure 1b). As multiple pili interact for generating bacterial motility, a two-state model for describing the time course of speed evolution was derived:

$$v(t)=v_{H}-\frac{v_{H}-v_{L}}{\exp\left(k\left(t_{gs}-t\right)\right)+1}$$ , (1)

where *t*_*gs*_ is the time point of global switching, and *k* is the rate at which the free energy difference between the states changes. The time point of global switching *t*_*gs*_ decreases inversely with the oxygen consumption rate (or concentration of cells) when the bacteria are the only consumers of oxygen in the sample [20]. We hypothesize that this multistate-system enables bacteria to tune T4P dynamics for rapidly responding to environmental conditions. However, the mechanism of oxygen-sensing is unclear.

Here, we investigated the influence of proton motive force (PMF), ATP-depletion, and nitrite on the speed of bacterial motility. Our data indicate that ATP is required for pilus retraction, but gonococci boost pilus retraction speed by a factor of two by using PMF as an additional energy source.

## Results

Oxygen is the final electron acceptor of the respiratory chain that helps maintaining the proton gradient between the cell’s interior and its exterior space. It was therefore conceivable that oxygen depletion correlated with depletion of the proton motive force. To our knowledge, the proton motive force (PMF) has not been characterized systematically in *N. gonorrhoeae* so far and thus we determined the PMF as a function of the external pH before addressing the question whether depletion of PMF triggered speed switching of gonococcal pili.

### Proton motive force of*Neisseria gonorrhoeae* at varying external pH

**Figure 2:**
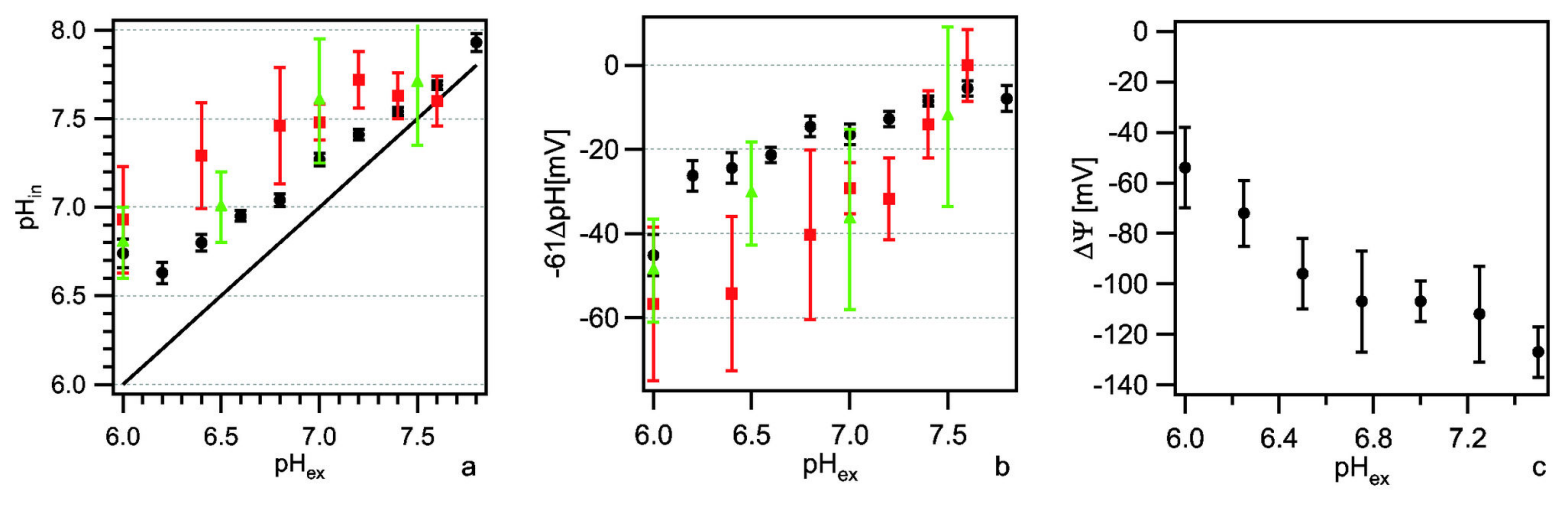
Components of the proton motive force of *N. gonorrhoeae*. (a) Intracellular pH (pH_in_) and (b) ΔpH-component of the PMF versus extracellular pH (pH_ex_). black: cFDA-SE (N = 100-1000 cells per data point), red: single cell analysis of *pHluorin* expressing cells (N = 240-530 cells per data point), green spectroscopic analysis of of *pHluorin* expressing cells (3 independent experiments). Full line in (a): pH_in_ = pH_ex_. (c) Transmembrane potential ΔΨ as a function of pH_ex_ (N = 100-200 cells per data point).

The proton motive force PMF = −61 · ΔpH + ΔΨ has two contributions, namely an entropic component (ΔpH) and an electrostatic component (ΔΨ). We determined both components using fluorescence microscopy at 37°C. For measuring the transmembrane pH difference, gonococci were loaded with the ratiometric pH-sensitive dye 5(6)-carboxyfluorescein diacetate N-succinimidyl ester (cFDA-SE). Calibration was performed as explained in the Methods S1 and Figure S1 in File S1. These experiments were conducted inside a flow cell during twitching motility assays. In all experiments availability of oxygen was verified by eye-inspection of twitching motility in high speed mode. The intracellular pH_in_ increased with increasing external pH (pH_ex_) (Figure 2a).

Since our results showed that homeostasis in *N. gonorrhoeae* is remarkably poor as compared to experiments with other bacterial species [21], we used a different method for determining pH_in_. We generated a gonococcal strain that expressed ratiometric *pHluorin*, a pH-sensitive GFP derivative [21] [22] (Figure S2 in File S1). Using ratiometric single cell fluorescence microscopy of the *pHluorin* expressing bacteria, we found slightly higher internal pH_in_ as a function of external pH_ex_ as compared to cFDA-SE (Figure 2 a, b). Since the calibration of the dye sensitively depends on full depletion of ΔpH, we compared our data with spectroscopic data where cells have been lysed for calibration and found good agreement (Figure 2 a, b). We conclude that although the absolute value measured for the internal pH_in_ is somewhat sensitive to the method used, pH homeostasis in the range of pH_ex_ 6 to 7.5 is not as pronounced as in other bacterial species such as *Escherichia coli*.

For determination of the transmembrane potential ΔΨ the cationic dye tetramethylrhodamine methyl ester (TMRM) was used. TMRM partitions between the cytoplasm and the exterior of the cell according to Boltzmann’s distribution and can be used to determine ΔΨ (Materials and Methods, Figure S3 in File S1). In agreement with the assumption that the ΔpH-component and the ΔΨ -component of the PMF can compensate each other, ΔΨ increased with decreasing ΔpH (Figure 2c and Table 1). These results demonstrate that the ΔΨ -component is highly dominant in retraction assay medium (RAM, pH 6.8) with a ΔpH-component −61 · ΔpH ≈ -30 mV and a membrane potential ΔΨ ≈ −110 mV.

**Table 1:** Bacterial strains.

**Strain**	**Parental strain**	**Genotype**	**Source of strain**
MS11	wt		
N400	MS11	*P*_*lac*_ * recA (tetM)*	[52]
pHluorin	N400	*iga::P*_*pilE*_ * pHluorin (erm)*	This study
DK1622	wt		

### Depletion of proton motive force triggers speed switching

**Figure 3:**
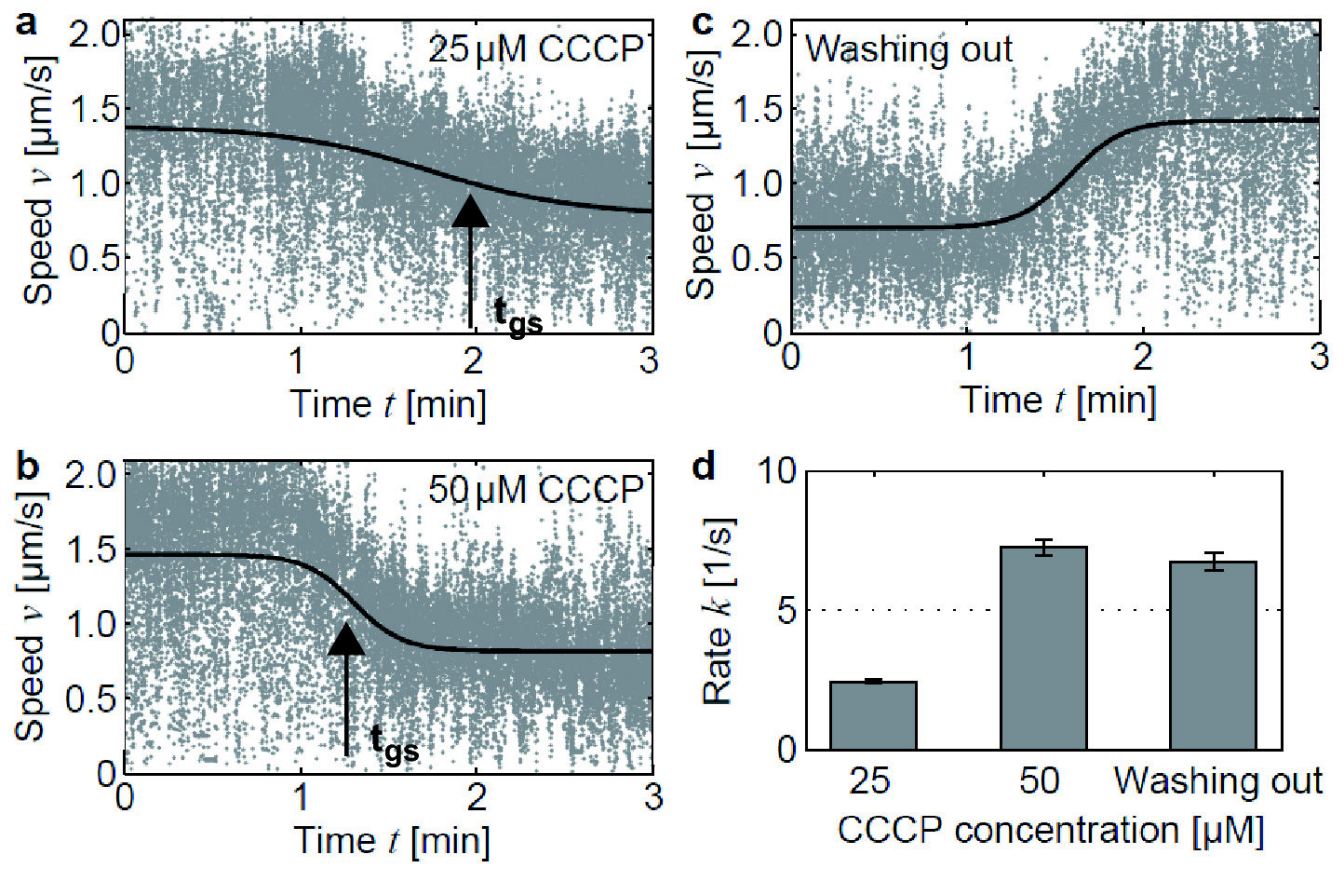
Depletion of proton motive force induces global switching and is fully reversible. (a) Global switching during injection of 25 µM CCCP. Overlay of speeds of 48 bacterial tracks versus time. Solid line: fit to eq. 1. (b) Global switching during injection of 50 µM CCCP. Overlay of speeds of 40 bacterial tracks. (c) Washing out CCCP is accompanied by switching back to high speed mode. Overlay of speeds of 35 bacterial tracks. (d) Transition rate as obtained by fit to eq. 1.

To understand the role of bioenergetics in oxygen-dependent speed switching, we investigated twitching motility in response to PMF depletion by application of carbonyl cyanide m-chlorophenyl hydrazone (CCCP). The protonophore CCCP is a potent uncoupler which depletes PMF on a time scale below 1 min at the concentrations we used [23] [24]. We conducted twitching motility assays inside a flow cell and injected CCCP at two different concentrations: 25 µM and 50 µM. In both cases, injection was followed by rapid speed switching, demonstrating that global switching can be induced by depletion of PMF (Figure 3a, b). The transition rate increased with increasing CCCP concentration. In addition, we proved that this process is fully reversible. After washing out CCCP, all bacteria switched back to the high speed mode within a time window of less than 60s (Figure 3c). The transition can be described using the two-state model eq. 1 (Figure 3d). This behavior is comparable to re-energizing cells after oxygen depletion with fresh oxygen-rich medium. The kinetics of switching from the high speed state to the low speed state is reminiscent of switching triggered by oxygen depletion [20]. This experiment is in agreement with the assumption that depletion of PMF is the underlying reason for speed switching in response to oxygen depletion and suggests that direct oxygen sensing is not involved.

### Inhibition of ATP synthase does not trigger speed switching of twitching motility

**Figure 4:**
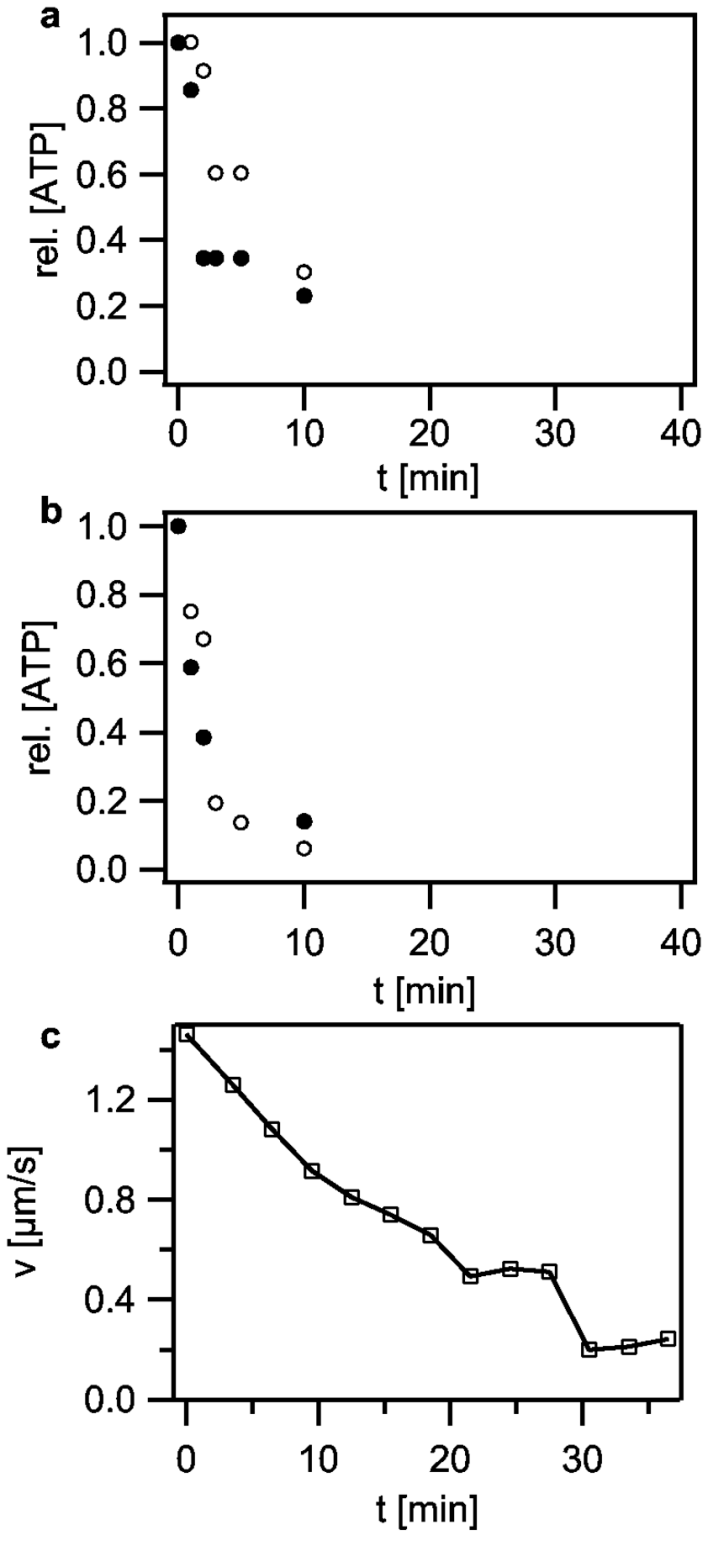
Effect of ATP depletion on twitching dynamics by direct inhibition of the ATP synthase. (a) Kinetics of ATP depletion upon treatment with 50 µM CCCP. Open and closed grey circles depict independent determinations of the relative ATP concentration, respectively. (b) Kinetics of ATP depletion upon treatment with 200 µM DCCD. Open and closed grey circles depict independent determinations of the relative ATP concentration, respectively. (c) Twitching speed averaged over 3 min versus incubation time in 200 µM DCCD.

Synthesis of ATP by the ATP synthase relies on the proton motive force. Therefore, we characterized the kinetics of decrease in ATP levels after treatment with 50 µM CCCP using a luciferase/luciferin assay (Figure 4a). The ATP concentration decreased considerably within minutes with a characteristic decay time of ≈ 5 min.

It was conceivable that the rapid decrease of ATP levels triggered speed switching. Thus, we applied the ATP synthase inhibitor N,N’-dicyclohexylcarbodiimide (DCCD) at 200 µM. The ATP decay kinetics was in agreement with an exponential function and the characteristic decay time was ≈ 2 min (Figure 4b). Next, we monitored twitching motility during treatment with DCCD. Again, these experiments were conducted inside a flow cell and twitching motility was directly monitored after DCCD injection. At 200 µM DCCD the twitching speed decreased very slowly (Figure 4c). The effect of DCCD on viability was very heterogeneous determined by twitching and cell appearance. The number of motile bacteria decreased drastically with increasing incubation time. However, fast speed switching, as induced by depletion of oxygen or PMF was not observed, even though ATP depletion occurred more rapidly as in the case of treatment with CCCP (Figure 3a,b). Below 100 µM DCCD, we observed no effect on twitching motility (Figure S4 in File S1). At 300 µM DCCD twitching speed decreased continuously until all bacteria stopped movement after ≈ 12 min of incubation (Figure S4 in File S1). We conclude that speed switching was not triggered by depletion of ATP.

### Depletion of ΔpH triggers speed switching and speed switching upon oxygen depletion is accompanied by reduction of ΔpH

**Figure 5:**
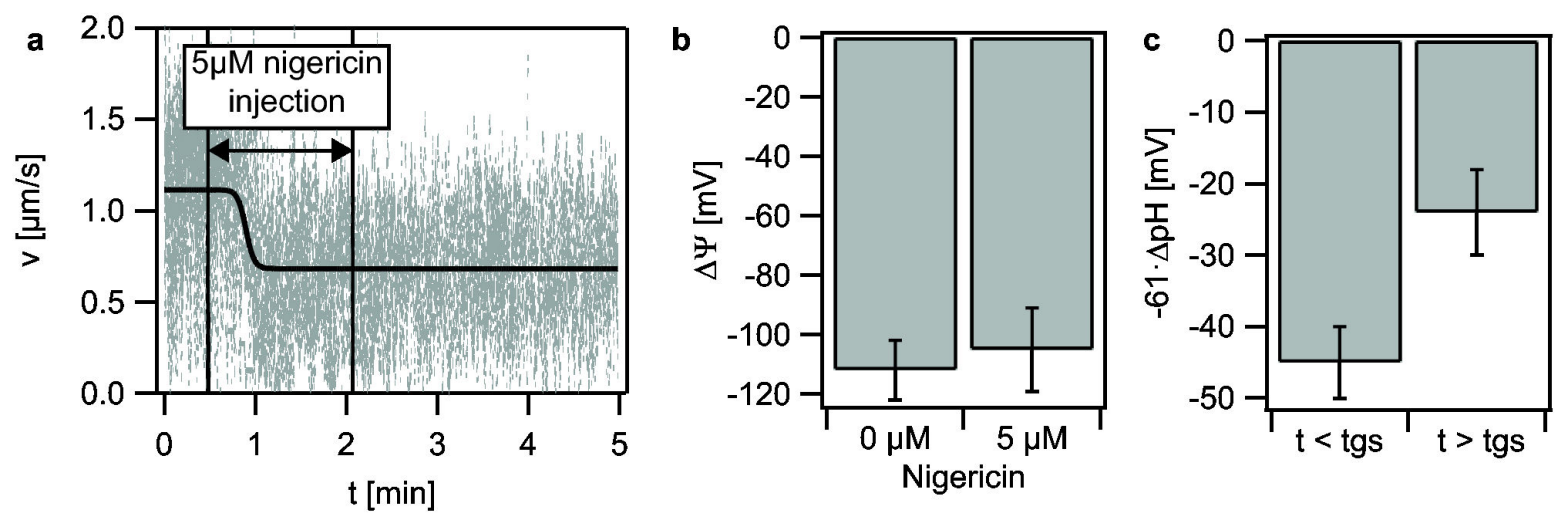
Global switching correlates with reduction of transmembrane ΔpH. (a) Addition of 5 µM nigericin induces global switching (overlay of 31 bacterial tracks). (b) Transmembrane potential ΔΨ before and after nigericin treatment. (c) −61·ΔpH before and after global switching induced by oxygen scavenger treatment at pH_ex_ = 6.0.

Nigericin is a H^+^–K^+^-antiporter and exclusively depletes ΔpH while maintaining ΔΨ. To monitor twitching motility during nigericin injection and to determine the membrane potential before and after drug treatment, we used a flow cell and loaded cells with TMRM. These experiments were conducted in RAM (pH 6.8) in which the ΔΨ -component of the PMF is dominant. Interestingly, application of 5 µM nigericin induced rapid speed switching (Figure 5a). If a single component of the PMF is depleted, e.g. by application of an ionophore, bacteria can rapidly upregulate the other component within several seconds up to a few minutes to maintain the PMF [23] [25]. We found that the membrane potential remained constant (Figure 5b). Thus assuming that the ΔpH was fully depleted, the reduction of PMF is only from PMF ≈ −140 mV before global switching to PMF ≈ −105 mV after global switching.

Next, we determined the ΔpH before and after global switching in response to oxygen depletion. Again, twitching motility assays inside a flow cell were performed and in this case cells were loaded with the pH-sensitive dye cFDA-SE. Because ΔpH was highest at pH_ex_ 6.0, we adjusted the medium to pH_ex_ 6.0 to obtain a significant effect. Global switching was induced by application of an oxygen scavenger system based on 2.5 mM protocatechuic acid (PCA) and 50 nM protocatechuate 3,4-dioxygenase (PCD) as described elsewhere [20]. Since global switching is reversible, these experiments could be repeated in the same sample. We found an average ΔpH = 0.74 ± 0.08 in the high speed mode and a ΔpH = 0.40 ± 0.11 in the low speed mode (Figure 5c). Although significant, again the reduction in ΔpH was not very high.

To confirm that the important component for speed switching was the pH difference over the cell membrane and not the internal pH, we assessed whether we were able to see speed switching upon oxygen depletion at varying extracellular pH_ex_ which correlates with varying intracellular pH_in_ (Figure 2). We found that speed switching upon oxygen depletion occurred between pH_ex_ 6.0 and pH_ex_ 7.8. We conclude therefore, that changes of internal pH cannot trigger global switching.

Taken together, we demonstrated that depletion of ΔpH induces speed switching and that oxygen depletion and reduction of ΔpH are strongly correlated.

### Nitrite and global switching

In the absence of oxygen, nitrite is an alternative electron acceptor for the respiratory chain in *N. gonorrhoeae* [26]. The ability of gonococci to reduce nitrite is regulated by the master regulator FNR whose expression is upregulated in the presence of nitrite after oxygen depletion. We tested whether addition of nitrite to RAM medium caused the cells to switch back to the high speed mode.

Following Falsetta et al. who found enhanced biofilm formation in the presence of 100 µM NaNO_2_ [27] [28], we conducted twitching motility assays inside enclosed chambers at the same concentration of nitrite in the absence of ascorbic acid. Higher concentrations of nitrite (10mM to 20mM) were also tested but showed a toxic effect determined by a strongly increasing number of non-motile cells with increasing incubation time.

The time point of global switching after oxygen depletion did not depend on the presence of nitrite. This was expected because denitrification must be induced by the oxygen-sensing transcription factor FNR [26] [29]. Although a few minutes after global switching a very small fraction of bacteria (< 5%) showed a twitching speed almost comparable to the high speed mode under aerobic conditions, addition of nitrite did not induce a back-switch of the population to the high speed mode within 1h after oxygen depletion.

### Mode switching is triggered by different inputs in *Myxococcus xanthus*

**Figure 6:**
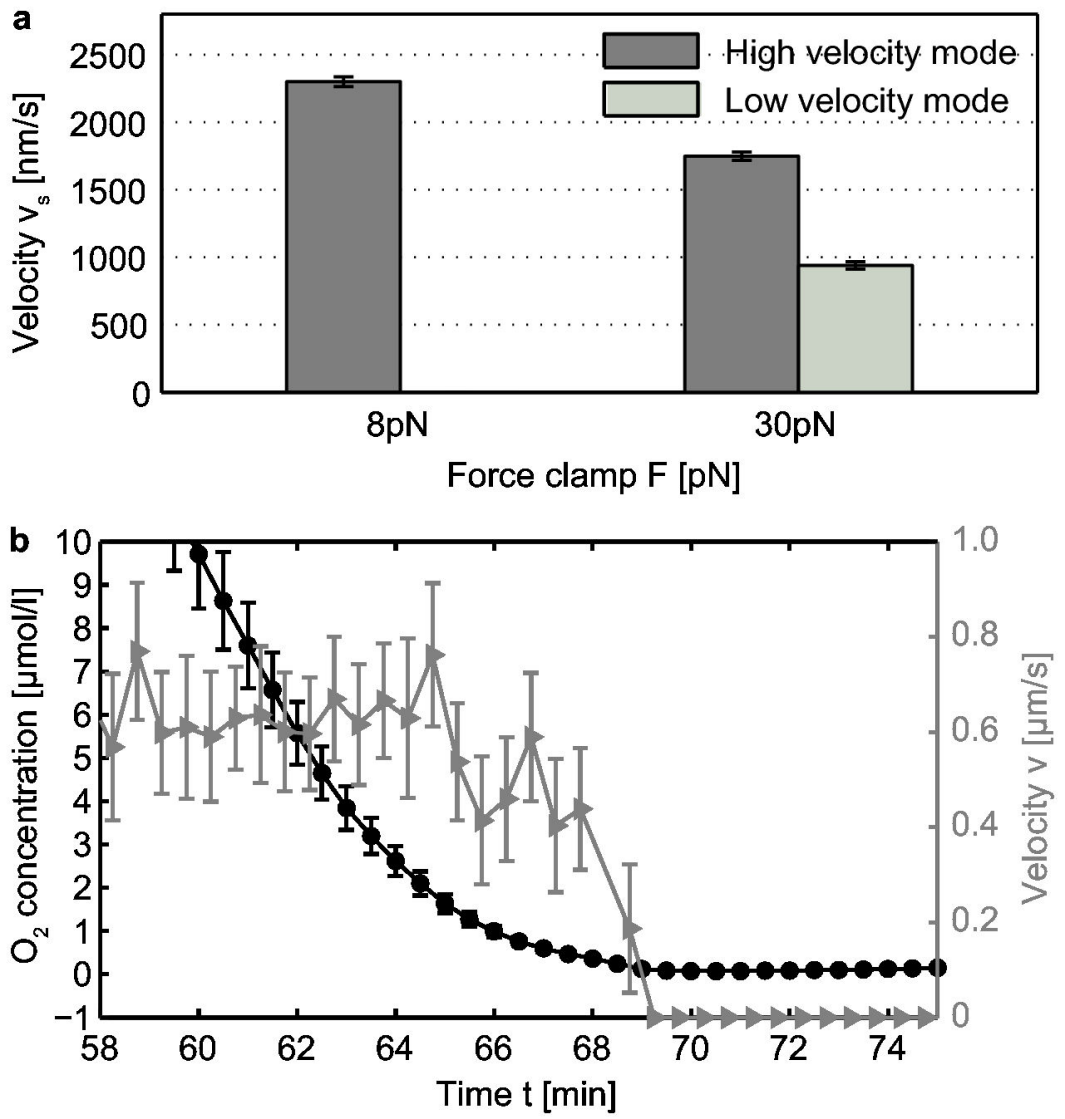
Oxygen-dependent mode switching is not conserved in *M. xanthus*. (a) Average speeds *v* of single pilus retraction at 8 pN (62 events) and 30 pN (56 events). (b) Oxygen concentration (black circles) and average speed *v* of moving bacteria (grey triangles, 5 bacteria per time point) as a function of time.

At the level of single pilus retraction, we have previously found that bimodality in speed occurred both in *N. gonorrhoeae* and in *M. xanthus* [19]. Here, we investigated pilus retractions of *M. xanthus* at a very low load of 8 pN, a force regime that we had not addressed in previous laser tweezers experiments. Interestingly, we found a dominant high velocity mode leading to a monomodal Gaussian distribution after averaging all 62 events with an average speed of v_s_ = (2300 ± 37) nm/s (Figure 6a). By increasing the clamp force to 30 pN, we observed frequent switching between the high and the low speed mode during single retraction events. This observation was independent of the time post sealing, i.e. of the oxygen concentration in the sample. In contrast, for *N. gonorrhoeae* we did not observe speed switching under aerobic conditions at force up to 60pN [20]. At very high force of 120pN, however, we detected speed switching even at high oxygen concentration (Kurre & Maier, unpublished).

Next, we applied the oxygen scavenger system to *M. xanthus*. We did not observe pilus retraction after incubation with the oxygen scavenger. To confirm that oxygen depletion affected motility in *M. xanthus*, we simultaneously measured the speed of surface motility and of oxygen concentration as demonstrated previously (details in [20]). We added a 50 µl cell suspension of *M. xanthus* to an oxygen sensor and sealed the sample. After attachment to the PDMS surface of the oxygen sensor, bacteria performed surface motility with an average speed v = (0.67 ± 0.01) µm/s (Figure 6b). At the time point of full oxygen depletion, motility was not detectable any more. Furthermore, we depleted PMF via 50 µM CCCP prior to sealing the sample. Again, pilus retraction was not detected.

Together, we have shown that although both speed modes exist in *M. xanthus*, switching between both modes is not triggered by oxygen depletion or depletion of PMF.

## Discussion

### Putative mechanisms of proton motive force induced speed switching

All of the evidence that we have obtained indicates that speed switching of gonococcal T4P retraction is triggered by depletion of PMF but not by ATP depletion. Our data is consistent with a picture in which the T4P system is in the high speed mode when the PMF is high and switches to the low speed mode upon reduction of PMF. We propose therefore, that the gonococcal T4P motor uses two sources of energy, namely ATP which supports the low speed mode and PMF for increasing the speed.

How does PMF couple to T4P retraction? One putative molecular mechanism would be that in addition to the retraction ATPase PilT another protein supports T4P retraction in a PMF dependent fashion. Furthermore, binding of regulatory proteins to the T4P complex may generate different states of pilus retraction. The PMF may be translated into motor speed through tuning the functionality of the motor by a regulating protein, reminiscent of the molecular brake in *E. coli* or the molecular clutch in *B. subtilis* interacting with flagellar rotation [30–33]. Another mechanism would involve PilT itself. PilT has a PAS-like N-terminal domain [7] that may be act as a sensor domain. PAS domains play a crucial role in sensing diverse physicochemical stimuli [34–36].

One possible mechanism for changing the molecular composition or conformation of the pilus motor would be a change of pH_in_. However, oxygen-dependent speed switching occurred at pH_ex_ ranging from 6.0 to 7.8, and changing pH_ex_ only influenced the oxygen depletion rate without affecting the absolute speed of the high and low speed mode. Since pH_in_ increased with increasing pH_ex_, a change of pH_in_ is unlikely to trigger mode switching.

### Contribution of the components of PMF

As it was expected from experiments in *B. subtilis*, *Escherichia coli*, or *Helicbacter* *pylori* [37–39], we have shown that pH_in_, ΔpH as well as ΔΨ are functions of pH_ex_ in *N. gonorrhoeae*. Consequently, ΔpH decreased with increasing pH_ex_, whereas ΔΨ increased to stabilize the PMF at changing pH_ex_. We conclude therefore, that our qualitative behavior for ΔpH as well as ΔΨ agree well with the literature values, even though pH homeostasis appears to be relatively poor. Please note that comparison of absolute bioenergetic parameters between different studies is quite challenging, since they strongly depend on experimental conditions such as surface charge [40].

### The role of the denitrification pathway in speed switching

It has been shown that *N. gonorrhoeae* can grow under anaerobic conditions in the presence of nitrite, using a truncated denitrification pathway. Anaerobiosis plays an important physiological role, since biofilm formation is accompanied by a transition to anaerobic conditions followed by anaerobic growth [28,41,42]. Therefore we addressed the question whether denitrification in the presence of nitrite can affect oxygen-dependent speed switching. Twitching motility assays revealed that the time point of global switching was unchanged. This was expected because denitrification must be induced by the oxygen-sensing transcription factor FNR [26] [29]. We did not observe a global switching back to the high speed mode even 60 min after the first global switching event, indicating that the denitrification pathway does not show a major influence on oxygen-dependent speed switching. Interestingly, both reductases of the truncated denitrification pathway, AniA and NorB, do not support PMF generation [43,44], indicating that PMF is significantly lower under exclusive nitrite respiration. Therefore, we hypothesize that the high speed mode, which is very likely coupled to the PMF by a so far unknown mechanism, cannot be driven by denitrification exclusively. We would like to point out, however, that we did not monitor the expression of *aniA* and *norB*, and therefore we cannot exclude that one or both genes may not have been induced under the conditions used in our experiments.

### Although the mechanical properties of the T4P motors are conserved, the trigger for speed switching is not

Interestingly, *M. xanthus* shows two retraction modes comparable in speed to *N. gonorrhoeae* but it shows a different response to oxygen depletion. Pilus retraction switches readily between both speed modes when forces exceed 8 pN, demonstrating that they are in a bistable regime under aerobic conditions. Similar bistability was found for *N. gonorrhoeae* only at forces exceeding 100 pN. Upon oxygen depletion, however, *M. xanthus* switched within minutes to a non-motile state, reminiscent of the oxygen response of *E. coli* [45]. Furthermore, pilus retractions were not detected after depletion of PMF via CCCP, indicating that depletion of PMF induces the switch to the non-motile state. Thus, speed switching in *M. xanthus* may be triggered by a so far undiscovered environmental input.

If reduction of cellular ATP levels and hence the occupation of the hexameric retraction ATPase PilT with ATPs would be exclusively responsible for speed switching, we expect that *M. xanthus* and *N. gonorrhoeae* responded in a similar way to cellular energy depletion. Recently, Sun et al. have shown that treatment of *M. xanthus* with nigericin does not affect T4P-dependent motility [46] in contrast to our results with *N. gonorrhoeae*. We conclude, therefore, that although the mechanical characteristics of T4P dynamics are well-conserved between bacterial species with different lifestyles, they have evolved to respond to different regulatory inputs.

In contrast to *M. xanthus*, which is an obligate aerobe, oxygen-dependent speed switching may have evolved in *N. gonorrhoeae* due to its oxygen-limited habitat in the human urogenital mucosa. In this scenario, *N. gonorrhoeae* switches to the low speed mode, which serves as a “power saving mode”, to save ATP for processes of vital importance when PMF is reduced and ATP is limited. This behavior could prevent energy depletion without needing to switch off T4P dynamics completely.

## Conclusion

Our experiments clearly demonstrate that reduction of proton motive force triggers speed switching of type IV pilus retraction to the low speed mode. Reduction of ATP levels does not trigger speed switching. We therefore suggest that the low speed mode of the pilus motor is exclusively driven by ATP-hydrolysis, whereas the high speed mode is caused by an additional coupling to the pH gradient/proton flux.

## Materials and Methods

### Bacterial strains, growth conditions, and media

*N* *. gonorrrhoeae* (Table 1) was grown overnight at 37°C and 5% CO_2_ on agar plates containing gonococcal base agar (10 g/l Bacto^TM^ agar (BD Biosciences, Bedford, MA, USA), 5 g/l NaCl (Roth, Darmstadt, Germany), 4 g/l K _2_HPO_4_ (Roth), 1 g/l KH_2_PO_4_ (Roth), 15 g/l Bacto^TM^ Proteose Peptone No. 3 (BD), 0.5g/l soluble starch (Sigma-Aldrich, St. Louis, MO, USA)) and the following supplements: 1g/l D-Glucose (Roth), 0.1 g/l L-glutamine (Roth), 0.289 g/l L-cysteine-HCL×H_2_0 (Roth), 1 mg/l thiamine pyrophosphate (Sigma-Aldrich), 0.2 mg/l Fe(NO_3_)_3_ (Sigma-Aldrich), 0.03 mg/l thiamine HCl (Roth), 0.13 mg/l 4-aminobenzoic acid (Sigma-Aldrich), 2.5mg/l β-nicotinamide adenine dinucleotide (Roth) and 0.1 mg/l vitamin B_12_ (Sigma-Aldrich). Before each experiment gonococcal colonies were resuspended in retraction assay medium (RAM) consisting of phenol red-free Dulbecco’s Modified Eagle Medium (GIBCO, Grand Island, NY, USA), 4.5 g/l Glucose (GIBCO), 2 mM L-glutamine (Roth), 8 mM sodium pyruvate (GIBCO), 5 mM ascorbic acid (Roth) and 30 mM HEPES (Roth). RAM had a final pH of 6.8. To adjust pH, RAM was titrated with HCl or sodium hydroxide.

To generate anaerobic conditions, an oxygen scavenger system based on 2.5 mM protocatechuic acid (Sigma-Aldrich, St. Louis, MO) and 50 nM protecatechuate-3,4-dioxygenase (Sigma-Aldrich) was added to the media (Aitken, 2008)

For *M. xanthus* wild type DK1622 [47] was used. Cells were grown in liquid medium or on 1.5% agar containing 1% CTT as described [48]. For experiments bacteria were grown to an optical density at 600nm of 0.3 at 32°C and 230 rpm and then directly transferred either to an oxygen sensor for twitching assays [20] or to a polystyrene coated glass slide for pilus retraction assays.

### Construction of *pHluorin* expressing strains

The *pHluorin* gene under the control of the *pilE* promoter was inserted into the *iga* locus of N400. The *pHluorin* sequence was amplified from the plasmid pGex, using the primers NK45 and NK52-SacI (Table S1 in File S1). The *pilE* promoter region was amplified from gDNA using the primers 83-SacI and NK44. The PCR fragments were fused and then cloned into the p2/16/1 plasmid using the SacI restriction site. Plasmid p2/16/1 is a derivative of pUP6 with a 4 kb fragment containing the gonococcal IgA1 protease gene (*iga*) and an *erm* ^*R*^ cassette. The plasmid with *pHluorin* gene was transformed into DH5α *E. coli* on 50µg/ml kanamycin LB plates and purified from *E. coli*.

The transformations in *Neisseria gonorrhoeae* were carried out with 500 ng of DNA and 500 µl of gonococcal cells (~ 5x10^8^ cells/ml) in GC broth plus supplements (see above) with 7 mM MgCl_2_, 1mM IPTG at 37 °C and 5% CO_2_. After 30 min of incubation cells were diluted 1:10 and further incubated for 3 h under constant shaking with 250 rpm before plating at different dilutions onto 2.5 µg/ml erythromycin agar plates. Using the twitching motility assay, we verified that *pHluorin* expressing cells were motile.

### Depletion and quantification of proton motive force

The PMF was depleted by adding carbonyl cyanide m-chlorophenyl hydrazone (CCCP, Sigma-Aldrich). CCCP stock solution (10 mM in DMSO) was stored at -20°C. To break down the transmembrane gradient, the K^+^–H^+^ antiporter nigericin (Invitrogen, Carlsbad, CA, USA) was used. Nigericin stock solutions (10mM in DMSO) were stored at -20°C. We verified, that DMSO added at the final concentrations used for the experiments, did not alter twitching motility.

The internal pH of *N. gonorrhoeae* was determined by the pH-sensitive flourophore 5(6)-carboxyfluorescein diacetate N-succinimidyl ester (cFDA-SE, Sigma-Aldrich) [25] [49]. Stock solutions (1 mM in DMSO) were stored at -20°C. Cells were loaded with 5µM cFDA-SE for 10 min in loading buffer (pH 8.0) containing 1x PBS (Roth), 30 mM HEPES (Roth) and 1 mM EDTA (pH 7.4, Roth). After uptake, cFDA-SE is hydrolysed by cellular esterases to 5(6)-carboxyfluorecein succinimidyl ester (cFSE) and subsequently conjugated to aliphatic amine functions [49]. A detailed protocol of the calibration can be found in Methods S1 in File S1. Unbound cFDA-SE was flushed out by washing with RAM.

Transmembrane potential Δψ of *N. gonorrhoeae* was measured by 0.1 µM tetramethylrhodamine methylester (TMRM, Sigma-Aldrich) [50]. Stock solutions (100 µM in DMSO) were stored at 4°C. Cells were pre-treated with 10 mM EDTA (pH 7.4, Roth) for 10 min before loading to increase membrane permeability for TMRM. A detailed protocol of the calibration can be found in Methods S1 in File S1.

### Fluorescence microscopy

Fluorescence measurements were conducted in epifluorescence mode at an inverted microscope (Eclipse TE2000, Nikon) equipped with a 120 W metal halogenide fluorescence lamp (X-Cite 120, EXFO), an EMCCD camera (Roper Cascade II:512, Photometrics), a computer-controlled motorized microscope stage (H117 ProScan, Prior Scientific) and a perfect focus system (T-PFB, Nikon). Two diﬀerent 100 x oil immersion objectives were used: a CFI Plan Fluor objective (NA 1.3, Nikon) for PtTFPP (oxygen) and cFDA-SE (*∆*pH) and a CFI Apochromat TIRF series objective (NA 1.49, Nikon) for TMRM (*Δψ*). To equilibrate samples at 32°C (*M. xanthus*) or 37°C (*N. gonorrhoeae*) the microscope stage was incorporated into a heated thermal insulation box. Fully automated acquisition and device control was achieved by imaging software (NIS-Elements AR, Nikon instruments Inc.). More detailed methods may be found in the Supplementary information.

### Twitching motility assays

Twitching motility assays of *N. gonorrhoeae* were performed on bovine serum albumin (BSA, Sigma-Aldrich) coated glass in a commercial microscope equipped with a heated thermal insulation box equilibrated at 37 °C. Bacterial motility was monitored via standard video microscopy (10 frames per second) and subsequent cell tracking in MATLAB R2009b (MathWorks Inc.). Tracking is based on the algorithms of J.C. Crocker and D. Grier originally written in IDL and transferred to MATLAB code by D. Blair and E. Dufresne [51]. (Download: http://physics.georgetown.edu/matlab/.) Applying this tracking algorithm to long rod-shaped *M. xanthus* cells was not successful. In this case, cells were manually tracked within the image processing software ImageJ.

### Single pilus retraction assays

Single pilus retraction events were measured with an optical tweezers in force clamp mode [16]. Bacteria were fixed to polystyrene spin-coated glass slides. Carboxylated polystyrene microspheres (Molecular Probes, Eugene, Oregon, USA) with 2 µm in diameter were added to cell suspension before sealing the chamber.

## Supporting Information

File S1 Supporting files. Methods S1. Table S1, Primers used in this study. Figure S1, Measurement of ΔpH using cFDA-SE. Figure S2, Calibration of pHluorin expressing cells. Figure S3, Correction for point spread function for determination of ΔΨ. Figure S4, Effect of different DCCD concentration on twitching dynamics.
(PDF)
