# Supplementary material for: Speed Switching of Gonococcal Surface Motility Correlates with Proton Motive Force
Source: PLoS One. 2013 Jun 24;8(6):e67718. doi: 10.1371/journal.pone.0067718 (PMC3691265; doi:10.1371/journal.pone.0067718)
Supplement: File S1 — Methods S1. Table S1, Primers used in this study. Figure S1, Measurement of ΔpH using cFDA-SE. Figure S2, Calibration of pHluorin expressing cells. Figure S3, Correction for point spread function for determination of ΔΨ. Figure S4, Effect of different DCCD concentration on twitching dynamics. (PDF) [file pone.0067718.s001.pdf]

Supporting methods for

Speed switching of gonococcal surface motility correlates with proton motive force

Rainer Kurre, Nadzeya Kouzel, Kanimozhi Ramakrishnan, Enno R. Oldewurtel & Berenike Maier

Department of Physics and Biocenter, University of Cologne, Germany

Twitching motility assays.

Twitching motility assays of *N. gonorrhoeae* were performed on bovine serum albumin (BSA, Sigma-Aldrich) coated glass (24×24 mm, Roth). Prior to coating with BSA, glass slides were washed in 1 M KOH for 10 min to clean surfaces and to increase hydrophilicity. After thorough washing with Milli-Q and drying under a nitrogen stream, glass surfaces were incubated with a BSA solution (10 mg/ml in Milli-Q) for 30 min. Finally, glass slides were washed again with Milli-Q and dried with nitrogen. To construct enclosed chambers with a well-defined volume, 50 µl of cell suspension was added to the centre of a microscope slide. Small drops of vacuum grease (GE Bayer Silicones, Roth) were applied onto the corners of a BSA coated glass slide and acted as spacers. The BSA coated glass slide was slightly pressed to uniformly distribute the cell suspension. After positioning of the glass slide, edges were sealed with VALAP (1:1:1 mixture of vaseline (Roth), lanolin (Roth) and paraffin wax (Roth)). To exchange media during twitching motility assays, a homemade flow cell was mounted into the microscope. It consists of a polytetrafluoroethylene (PTFE) construction where a single channel (length: 56 mm; width: 6 mm; height: 1 mm; volume: 336 µl) is fabricated by adding a cover slide on top and a BSA coated glass slide at the bottom of this construction. Glass slides were sealed with VALAP. A single inlet and outlet allows for exchanging the medium by pipetting. Velocities $v_i(t)$ of single bacterial tracks were calculated by $v_i(t) = (r(t+\Delta T)-r(t))/\Delta T$, where $\Delta T = 1$ s and $r(t)$ is the position vector at time t.

Single pilus retraction assays using optical tweezers.

Sample chambers were constructed as described for twitching motility assays, but bacteria were fixed to polystyrene spin-coated glass slides (Roth, 24×24 mm) and the sample is mounted on a piezo stage (PI, Karlsruhe, Germany). Carboxylated polystyrene microspheres (Molecular Probes, Eugene, Oregon, USA) with 2 µm in diameter were added to cell suspension before sealing the chamber. To monitor a single pilus retraction event, an optically trapped microsphere, also called bead, was moved towards a single bacterium. The trapped bead was directly imaged onto a four-quadrant photo diode, monitoring the displacements

with high spatial and temporal resolution. The piezo stage was actuated by a computer-controlled force feedback loop to record pilus dynamics at constant force. For this, the displacement of the bead due to a pilus retraction was allowed to reach a certain threshold. Hereafter, the displacement and thus the force was kept constant by moving the piezo stage with the bacterium relative to the optical tweezers. The movement of the bacterium (piezo stage) is a direct measure of the pilus length change.The data was analyzed with software written in MATLAB R2009b (Math Works Inc.). Raw data was recorded with 20 kHz. Each event was smoothed with a nonlinear median filter using a moving window of 1 ms. Subsequently, data was down-sampled to a resolution of 10 ms for *N. gonorrhoeae* and 50 ms for *M. xanthus*, respectively. Velocities v were calculated by the difference quotient of down-sampled datasets. To account only for retraction periods, datasets were dissected into elongation ($v < -150$ nm/s), retraction ($v > 150$ nm/s) and pause periods with $|v| = 150$ nm/s. Retraction speed switching was identified by analyzing speed histograms and pilus length changes versus time of consecutively measured retraction events.

Simultaneous measurements of dissolved O_2 and motility.

Sealed chambers with a volume of 50µl equipped with the oxygen sensor were assembled as for twitching motility assays. PtTFPP phosphorescence and bacterial motility in brightfield mode were simultaneously monitored at the PDMS/medium interface of the oxygen sensor. PtTFPP signals were collected with a Cy3 filter set (EX: BP 535 ± 25 nm, BS: 580 nm, EM: LP 590 nm, Zeiss) and 100 ms integration time. The phosphorescence of PtTFPP is quenched by the presence of oxygen resulting in a strong effect on the viability of bacteria most likely due to generation of reactive oxygen species. To circumvent this problem the field of view (82×82 µm) was shifted by linearly scanning the sample with a step size of 200 µm. At each positions, surface motility of *M. xanthus* was recorded with a rate of 1 fps for a duration of 1 min followed by a single capture of PtTFPP phosphorescence. Thus, the oxygen measurement was not conducted at a single position, but monitored along a line through the entire sample. The scanning procedure was feasible because the distribution of cells was homogeneous and the response of the oxygen sensor only marginally depended on the position inside the sample.

Calibration: Phosphorescence of PtTFPP follows the Stern-Volmer equation:

$$\frac{I_0}{I} = 1 + K_{SV} \cdot [O_2]. \qquad (1)$$

Here, I is phosphorescence intensity, I_0 intensity in the absence of oxygen, K_{SV} Stern-Volmer constant and $[O_2]$ the oxygen concentration as previously described (Kurre & Maier, 2012). The phosphorescence of the entire field of view was averaged for each time point and the corresponding oxygen concentration was calculated by Eq. (1) with $K_{SV} = (0.70 \pm 0.09)$ l/µmol and $I_0 = 27\,896 \pm 545$.

Measurement of transmembrane pH gradients with cFSE.

The fluorescence of cFSE was measured in flow cells during twitching motility assays with a Yellow GFP emission filter (HQ 535 ± 30 nm, AHF) and a 100 ms integration time. Excitation of pH-insensitive wavelength (~ 440 nm) was achieved by a Cyan GFP excitation filter (D 436 ± 10 nm, AHF) and a 450 nm beam splitter (450 DCLP, AHF), excitation of pH-sensitive wavelength (~ 490 nm) by a Yellow GFP excitation filter (HQ 500 ± 20 nm, AHF) and a 515 nm beam splitter (Q 515 LP, AHF) (Fig. 6a). Supplementary figure S1a illustrates the fluorescence signals of a single cell after excitation at 490 nm and at 440 nm, respectively. Via automated multipoint acquisition fluorescence of 50-150 single cells were captured for each excitation wavelength at at least 6 different positions in the sample. Single cell fluorescence was analysed by routines written in MATLAB. Following the analysis of Lo et al. (2), the total single cell fluorescence intensities F_{440nm} and F_{490nm} were calculated by

$$F = \langle I - I_{bg} \rangle_{I>T_{cell}}, \qquad (2)$$

where I is pixel intensity, I_{bg} average background intensity around the cell (window 30×30 pixels) and T_{cell} a well-defined threshold to account mostly for intracellular fluorescence. I_{bg} was calculated by fitting a Gaussian function, $g(I) = a \cdot \exp\left(-\left(I - I_{bg}\right)^2/b^2\right)$, to the pixel intensities in the window around the cell. The threshold T_{cell} was defined by $T_{cell} = (I_{max} - I_0)/2$, where I_{max} is maximum pixel intensity and I_0 the smallest I for which $I > I_{bg}$ and $g(I) < 1$. The final measure was the ratio $R = F_{490nm}/F_{440nm}$. The latter had to be calibrated. Therefore, the PMF was depleted by 30 µM nigericin and 5 µM valinomycin (incubation time ~ 30 min) to guarantee that $pH_{ex} = pH_{in}$. Under these conditions, the calibration curve shown in supplementary Fig. S1b was obtained. This calibration curve could be used to determine ΔpH at external pH ranging from pH_{ex}=6.0 up to pH_{ex}=7.8.

Measurement of transmembrane pH gradients with individual *pHluorin* expressing cells.

The cells expressing *pHluorin* were immobilized on the polystyrene spin-coated glass slides and observed in the sealed chambers with a volume of 50µl. Fluorescence of fixed cells was observed using excitation wavelengths of 410nm (excitation increases as pH increases), achieved by BrightLine HC 406/15 excitation filter (AHF) and 470nm (excitation decreases as pH increases), achieved by a Yellow GFP excitation filter (HQ 500 ± 20 nm, AHF) and a 515 nm beam splitter (Q 515 LP, AHF). Emission was observed at Yellow GFP emission filter (HQ 535 ± 30 nm, AHF) and a 100 ms integration time. Via multipoint acquisition fluorescence single cells were captured for each excitation wavelength at a minimum of 6 different positions in the sample.

Single cell fluorescence was analysed by routines written in MATLAB. Windows (30x30 pixels) with single bacteria were found. Using the isodata algorithm (Ridler & Calvard, 1978)

on the combined fluorescence of both the 410nm and 470nm excited window, an automatic threshold was found and the same pixel mask was assigned to both wavelengths. The average fluorescence inside the pixel mask was obtained for each excitation and corrected for background intensity, which was calculated by fitting a Gaussian function on the pixel window. In a separate sample the same analysis was done on wild-type cells, which do not express *pHfluori*n, to obtain an estimate of the auto-fluorescence of the cells. After deduction of this auto-fluorescence estimate, the corrected intensity values were used to compute the 410/470 fluorescent ratio of the cell inside the window. The standard curve was obtained by fitting a sigmoid function to the fluorescent ratios obtained with a collapsed *ΔpH*. This fit was then used to convert the fluorescent ratios to internal pH values.

For calibration (Fig. S2a) cells were preincubated for 10 min with 10mM EDTA to increase membrane permeability for nigericin. Afterwards ΔpH was collapsed by the addition of 50 μM nigericin and 40 mM methylamine hydrochloride with the incubation time ~ 50 min at 37°C.

Measurement of transmembrane pH gradients with *pHluorin* expressing cells using fluorescence spectroscopy.

Fluorescence excitation spectra of radiometric *pHluorin* in different pH-adjusted buffers were determined with a spectrophotometer (Perkin Elmer LS55) (Fig. S2b). The buffers used had the concentration of 30mM MES (pH5.5), MOPS (pH6, pH6.5), and HEPES (pH7, pH 7.5) . The pH was determined after enriching each of these buffers with 11mM glucose and 8mM sodium pyruvate. For calibration, *pHluorin* expressing cells were adjusted to an OD_{600} 10.0 and sonicated (Fig. S2c). Samples with defined pH were excited at between 350nm and 500nm and the fluorescence emission was collected at 510nm. Spectra in Fig. S2b were normalized to their isobestic point at 428 ± 5nm. For background correction, fluorescence collected from N400 cells in the respective buffers was substracted. For determination of pH_{in}, *pHluorin* expressing cells were treated as described but not sonicated. Data from three independent experiments were averaged.

Quantification of membrane potential Δψ.

Due to the membrane potential Δψ, a cationic dye such as TMRM equilibrates across the membrane according to Boltzmann's law:

$$C_{in}/C_{ex} = exp(-q\Delta\psi/k_B T), \quad (3)$$

where C_{in}/C_{ex} is ratio of intracellular and extracellular TMRM concentration, q charge of TMRM, k_B Boltzmann's constant and T absolute temperature. The ratio C_{in}/C_{ex} could not be directly derived from raw fluorescence data F_{in}/F_{ex} because diffraction of single cell fluorescence would lead to an underestimation of C_{in}. Therefore, a correction factor $S(F_{in}/F_{ex})$ was derived from a three dimensional convolution model (Lo *et al.*, 2007):

$$C_{in}/C_{ex} = S(F_{in}/F_{ex}) \cdot F_{in}/F_{ex}. \quad (4)$$

Experimental point spread function: For the convolution model the experimental point spread function $PSF(x_i, y_i, z_i)$ was determined by single 20 nm fluorescence microspheres (FluoSpheres, Molecular Probes, Ex. 580 nm, Em. 605 nm), which were fixed to a cover glass. The motorized stage allowed for precise scans in z-direction with a step size $\Delta z = 160$ nm and $z_{max} = \pm 2.4$ µm regarding the centre of the fluorescence spheres. With a pixel size of 160nm a cubic grid of the PSF was obtained. For the convolution model the PSF was background corrected and normalized. Photobleaching was negligible. Supplementary Fig. S3a shows the PSF at the centre of the sphere and at extreme z positions.

Convolution model: N. gonorrhoeae cells appear as mono- or diplococci. The diameter of these spherical cocci is 0.5-1.0 µm determined by transmission electron microscopy (3). Therefore, gonococci were modelled by a sphere with 750 nm in diameter (monococcus) or with two spheres side by side (diplococcus), which stuck to a cover glass. Because a microscopic image had a pixel size of 160 nm, the radius of the sphere was only about two pixels in size. In this model, the TMRM concentration $C = (x_i, y_i, z_i)$ is distributed as follows:

$$C(x_i, y_i, z_i) = \begin{cases} 0, \text{ for } z \leq 0 \text{ (glass)}, \\ C_{ex} \text{ for medium}, \\ C_{in} \text{ for cell}, \end{cases} \quad (5)$$

where i indicates the corresponding voxel in a cubic grid with an edge length of 160 nm. Using the experimental PSF, a modelled blurred image $I_m(x_j, y_j, z_0 = 0.375 \mu m)$ of $C = (x_i, y_i, z_i)$ at midcell position was calculated by convolution:

$$I_m(x_j, y_j, z_0) = \sum_i [C(x_i, y_i, z_i) \cdot PSF(x_j - x_i, y_j - y_i, z_0 - z_i)], \quad (6)$$

This image I_m was the input for Eq. (2) to calculate the internal fluorescence F_{in} with $I_{bg} = 0$. The external fluorescence was determined by the averaged pixel intensity with $C_{in} = C_{ex}$. Supplementary Fig. S3b illustrates the real intensity profile $I_r(x, y, z = \text{midcell})$ before diffraction, the modelled blurred image $I_m(x, y, z = \text{midcell})$, and a binary image of all pixels above threshold defined by Eq. (2). By varying the ratio C_{in}/C_{ex}, the correction factor S as a function of F_{in}/F_{ex} was calculated by Eq. (4) (Supplementary Fig. S3c). Finally, Eq. (3) enabled the determination of the transmembrane potential $\Delta\psi$ for a measured ratio F_{in}/F_{ex} (Supplementary Fig. S3d). Due to the marginal difference between the curves for modelled mono- and diplococci, the transmembrane potential of single gonococci was analysed with an averaged correction factor $S(F_{in}/F_{ex})$ without considering appearance of cells. The goodness of this convolution model and the fact that single cell fluorescence was collected from moving cells during twitching motility assays should influence the uncertainty of $\Delta\psi$ measurements. To account for these error sources, the error of $\Delta\psi$ was estimated by the standard deviation and not, as usual, by the standard error.

Experimental procedure: Fluorescence signals were collected with a Cy3 filter set (Excitation: BP 535 ± 25 nm, beam splitter: 580 nm, emission: LP 590 nm, Zeiss) 30 min after loading with TMRM (conducted in a flow cell as for twitching motility assays). Via automated multipoint acquisition 100-200 single cells were captured with 100 ms integration time at midcell position at ten different positions in the sample. For each cell, the total single cell fluorescence F_t was determined by Eq. (2) and averaged to obtain $\langle F_t \rangle$. Furthermore, the average background intensity $\langle I_{bg} \rangle$ of all cells was calculated, which was proportional to the external fluorescence F_{ex}. To account for membrane bound TMRM fluorescence F_m, cells were treated with 50 µM CCCP for 30 min to deplete $\Delta\psi$ and a second multipoint acquisition was performed to acquire $\langle F_m \rangle$. In a last step, TMRM was flushed out by thoroughly washing with RAM and the average background $\langle I_0 \rangle$ was measured. Then, F_{ex} was determined by $F_{ex} = \langle I_{bg} \rangle - \langle I_0 \rangle$. Finally, the internal fluorescence due to free TMRM was estimated as $F_{in} = \langle F_t \rangle - \langle F_m \rangle + F_{ex}$.

Determination of ATP depletion kinetics.

ATP measurements were performed with an ATP determination kit (A22066, Invitrogen). To check for linearity, ATP standard solutions were evaluated for ATP dilutions ranging from 25nM, 100nM, 200nM before each measurement. Bioluminescence was quantified using a luminescence spectrometer (Perkin Elmer LS55). Bacteria were suspended in RAM medium and the optical density was adjusted to 1.0, centrifuged (13,000g, 1min), and frozen for 5 min. Subsequently, cells were suspended in resuspension buffer (60mM HEPES-Roth, 2x PBS-Roth) with GT buffer (40% glucose - Roth, 0.8% Triton X 100-Roth) and kept at room temperature for 10 min. The optical density was measured at this point for reference. After 5 min of incubation 5µl buffer T (200 mM HEPES, 8 % v/v Trition X-100) were added to 95 µl of buffer M (A22066) and incubated for 5 min. 10 µl of cell suspension were added and luminescence was measured after 1 min of incubation. The same procedure was followed for the gonococcal cells treated with CCCP (50 µM) or DCCD (200 µM).

Kurre, R. & B. Maier, (2012) Oxygen depletion triggers switching between discrete speed modes of gonococcal type IV pili. *Biophys.J., accepted*.

Lo, C. J., M. C. Leake, T. Pilizota & R. M. Berry, (2007) Nonequivalence of membrane voltage and ion-gradient as driving forces for the bacterial flagellar motor at low load. *Biophys J* **93**: 294-302.

Ridler, t. & s. Calvard, (1978) Picture thresholding using an iterative selection method. *IEEE Trans. System, Man and Cybernetics* **SMC-8**: 630-632.

Supporting tables

Table S1 Primers used in this study

Primer	*Sequence*
83-SacI	5’-TTGAGCTCTTCCGACCCAATCAACACACCCGATAC-3’
NK45	5’-AAGTTCTTCTCCTTTACTCATAAAATTACTCCTAATTG-3’
NK44	5’-TTCAATTAGGAGTAATTTTATGAGTAAAGGAGAAGAAC-3’
NK52-SacI	5’-TTGAGCTCTTATTTGTATAGTTCATCCATGCCATGTGTAATCCCAGCAGCTGTTACAAACTCAAGAAG-3’

Supporting figures

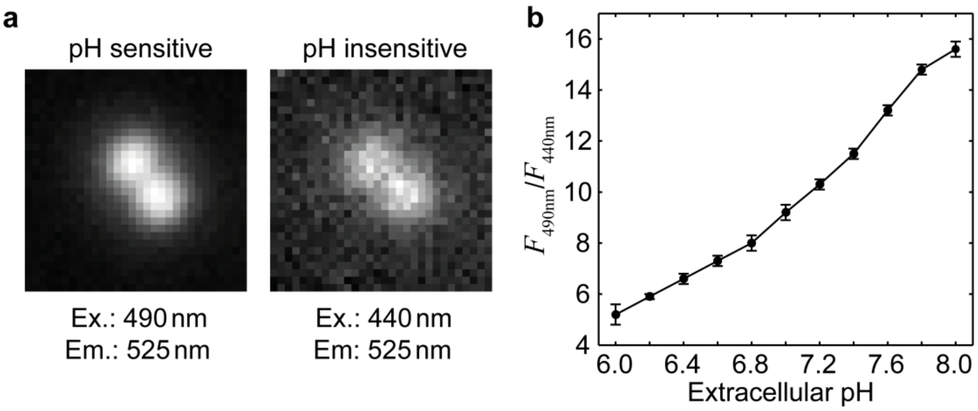

Supporting Fig. S1: Measurement of ΔpH using cFDA-SE. a) Fluorescence intensity *F* of a single diplococcus for emission at 525nm and excitation at 490nm (left) and 440nm (right). b) Calibration curve of cFSE. Ratio of fluorescence intensities F_{490nm}/F_{440nm} as a function of extracellular pH in the presence of 5μM valinomycin and 30μM nigericin (each data point averaged over 500-1000 cells).

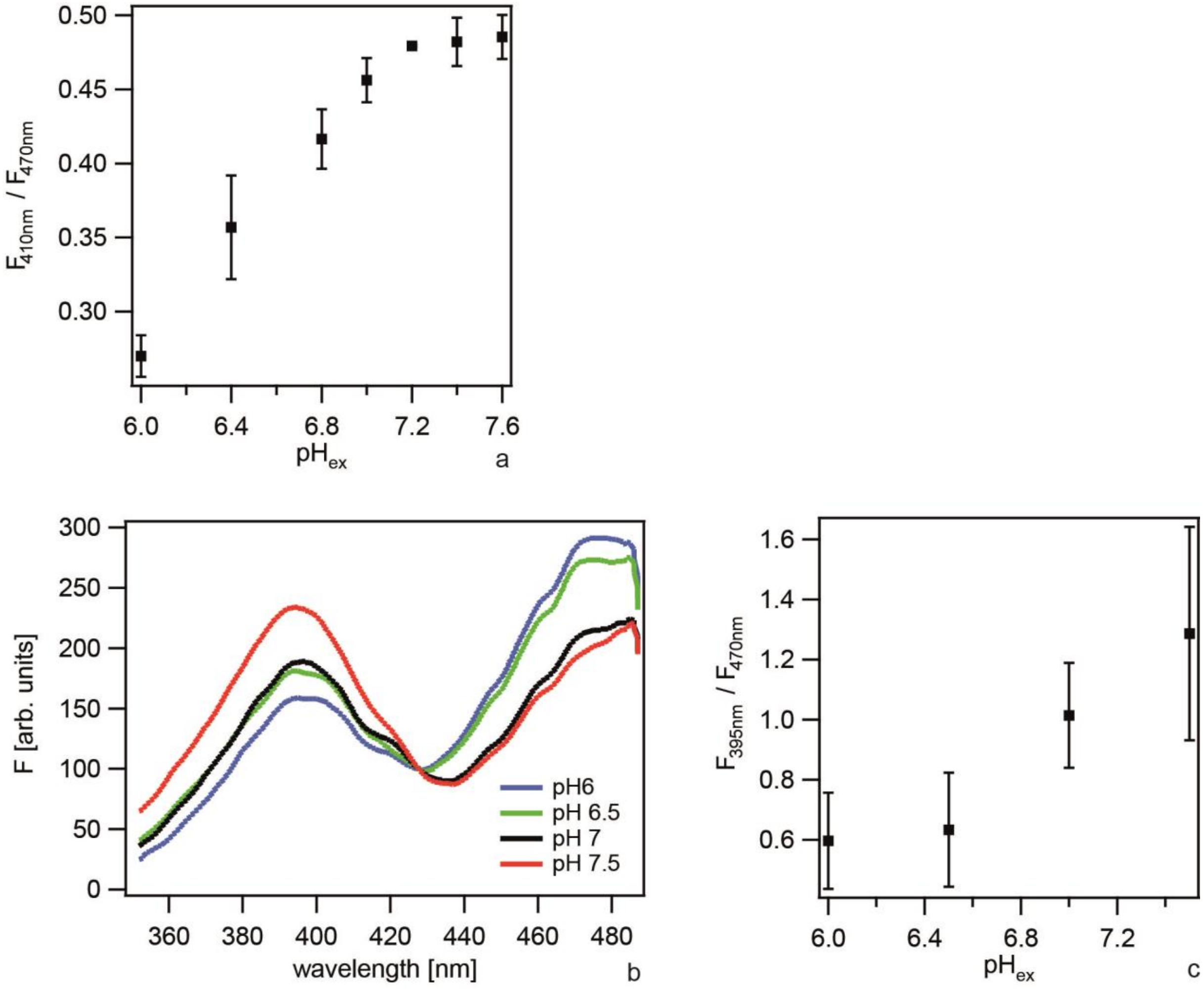

Supporting Fig. S2: Calibration of *pHluorin* expressing cells. a) Calibration curves of *pHluorin* expressing cells using single cell analysis. Each data point is averaged of 140 - 500 cells. b) Spectrum of *pHluorin* expressing gonococci after lysis at varying pH. Average of 3 independent data sets. c) Calibration curves of lysed *pHluorin* expressing cells using a fluorescence spectrometer. Average and standard deviation of three independent data sets. Please note that the filter set used for microscopic single cell analysis recorded a different ratio as the fluorescence spectrometer.

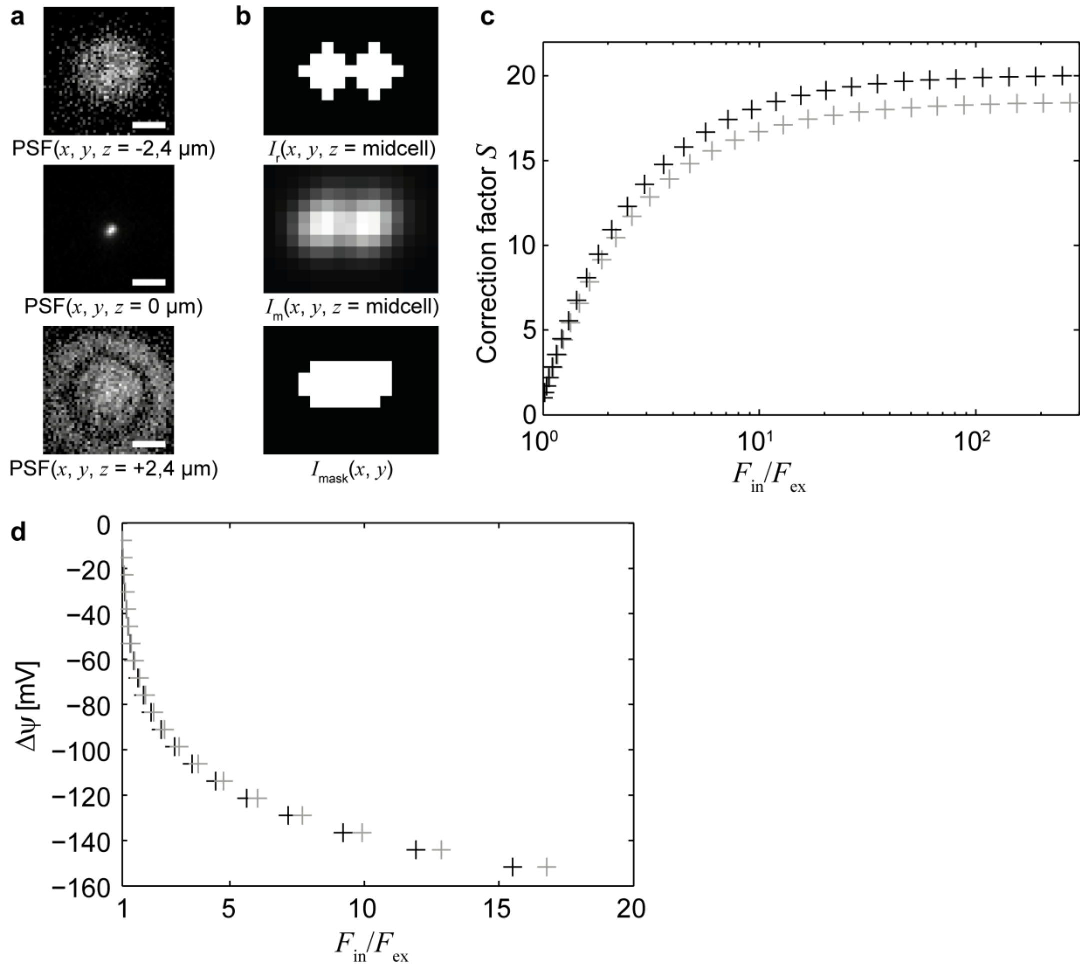

Supporting Fig. S3: a) Experimental point spread function (PSF) at z = -2.4 µm (below focus plane), z = 0 µm (midcell position) and z = +2.4 µm (above focus plane). Scale bar 2 µm. b) Convolution model for single cell fluorescence. Top: real intensity profile I_r of a diplococcus at midcell position modelled by two spheres with 750 nm in diameter. Pixel size 160 nm. Mid: I_m is corresponding intensity profile after convolution with experimental PSF. Bottom: I_{mask} is a binary image illustrating all pixels above the intensity threshold defined by Eq. (3). c) Correction factor S versus measured ratio of internal and external TMRM fluorescence F_{in}/F_{ex} for modelled monococci (black crosses) and diplococci (gray crosses). d) Corresponding transmembrane potential Δψ versus F_{in}/F_{ex} calculated by Eq. (4) and (5).

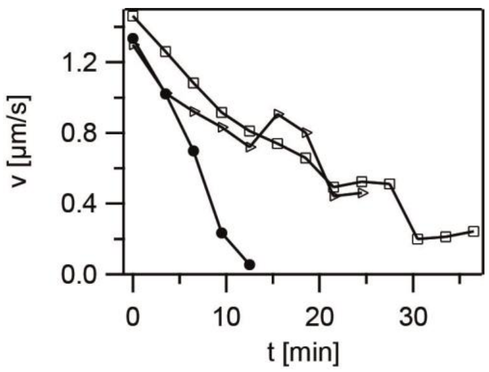

Supporting Fig. S4: Effect of ATP depletion on twitching dynamics by direct inhibition of the ATP synthase. Twitching speed averaged over 3 min versus incubation time in 100 µM DCCD (open triangles), 200 µM DCCD (open squares) and 300 µM DCCD (solid circles).
